# Supplementary material for: Evaluation of GPT and BERT-based models on identifying proteinprotein interactions in biomedical text
Source: ArXiv. 2023 Dec 13:arXiv:2303.17728v2. Preprint. [Version 2] (PMC11101131)
Supplement: 1 [file NIHPP2303.17728V2-supplement-1.pdf]

Supplementary Figures

Supplementary Figure 1. PPI mentioned in a biomedical text.

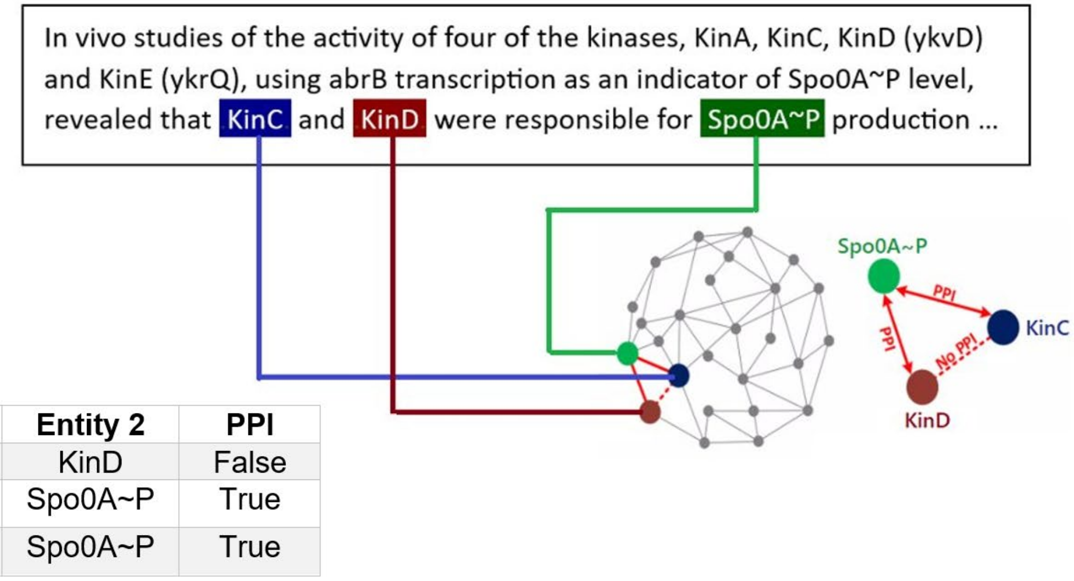

Entity 1	Entity 2	PPI
KinC	KinD	False
KinC	Spo0A~P	True
KinD	Spo0A~P	True

Supplementary Figure 2. Overview of methodology.

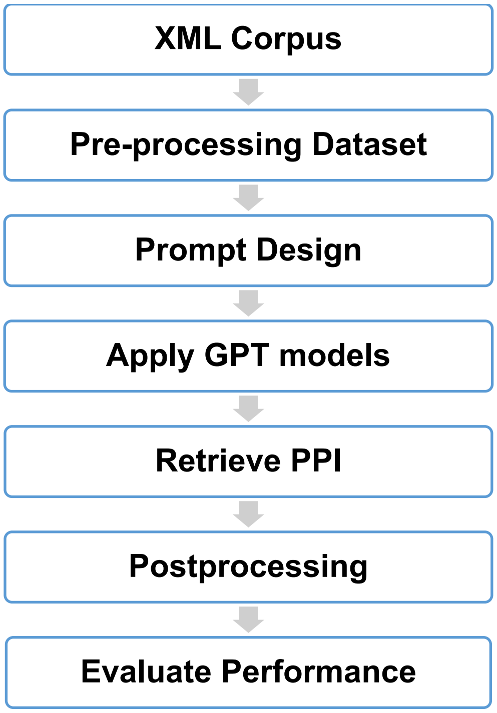

Supplementary Figure 3. Prompt Engineering for Base Prompt- Section 1

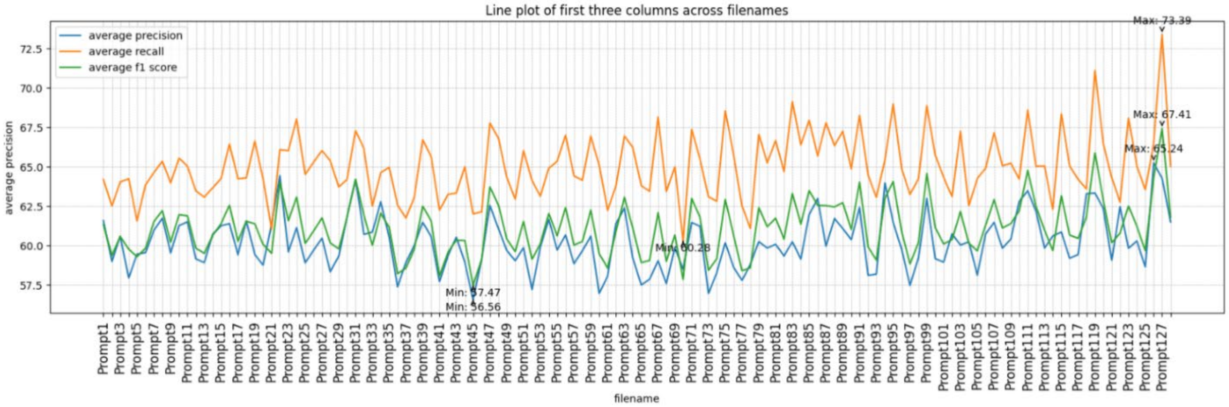

Supplementary Figure 4. Prompt Engineering for Base Prompt- Section 2

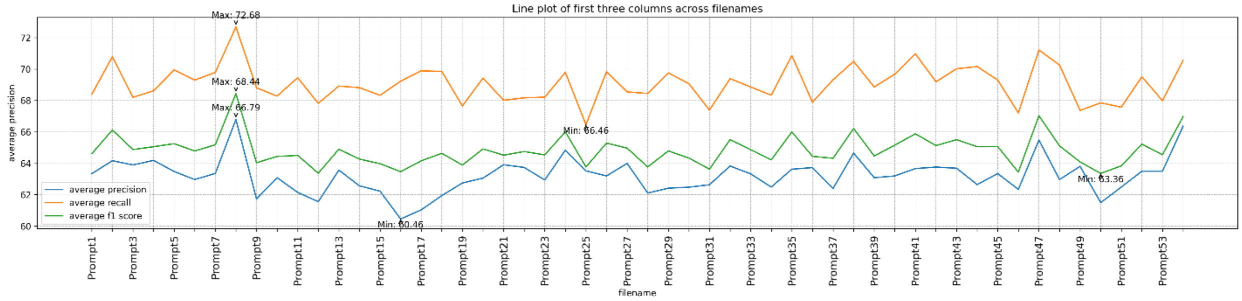

Supplementary Figure 5. Prompt Engineering for Base Prompt- Section 3

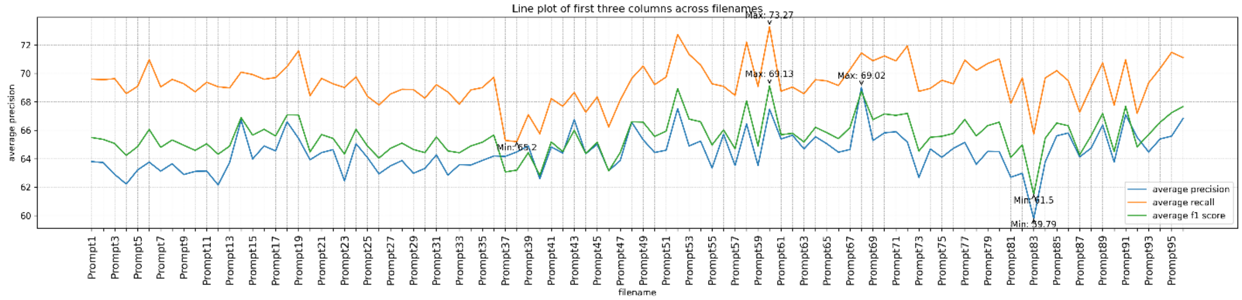

Supplementary Figure 6. Prompt Engineering for Base Prompt- Section 4

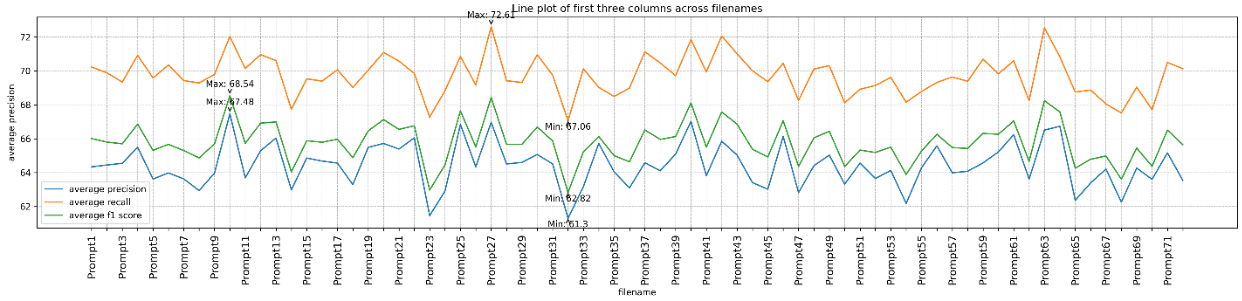

Supplementary Figure 7. Prompt Engineering for Base Prompt- Section 5

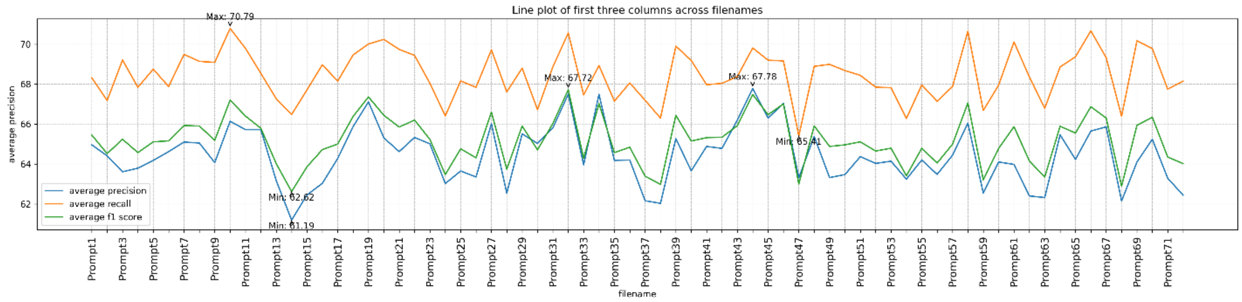

Supplementary Figure 8. Prompt Engineering for Base Prompt- Section 6

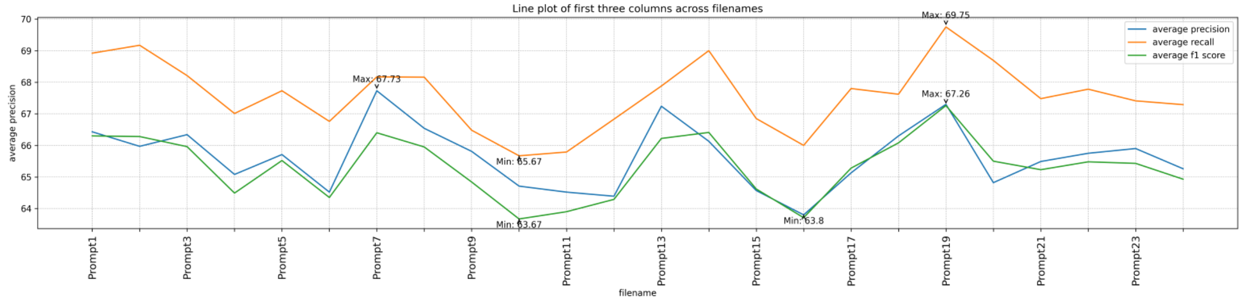

Supplementary Figure 9. Prompt Engineering for Base Prompt- Section 7

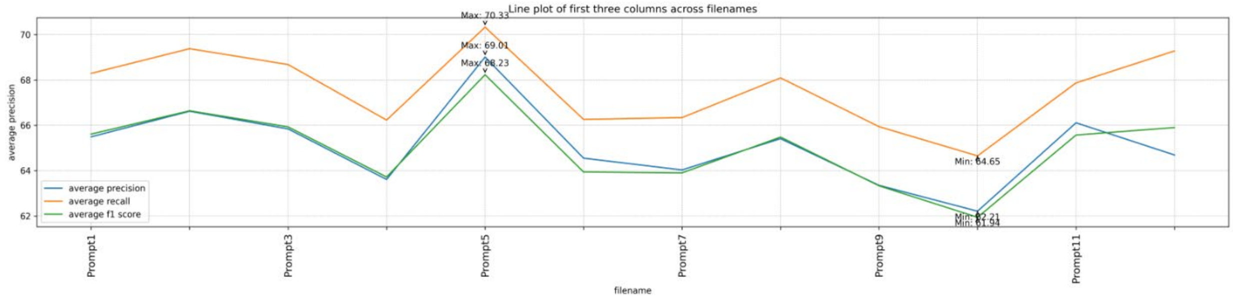

Supplementary Figure 10. Prompt Engineering for PROTEIN masked Prompt

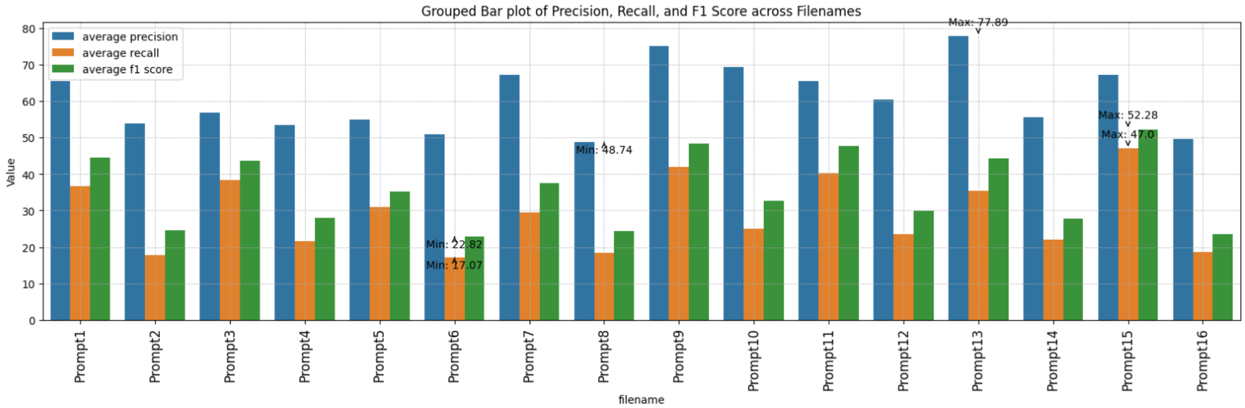

Supplementary Figure 11. Evaluation result of PPI identification on LLL dataset for BERT and GPT Based Models

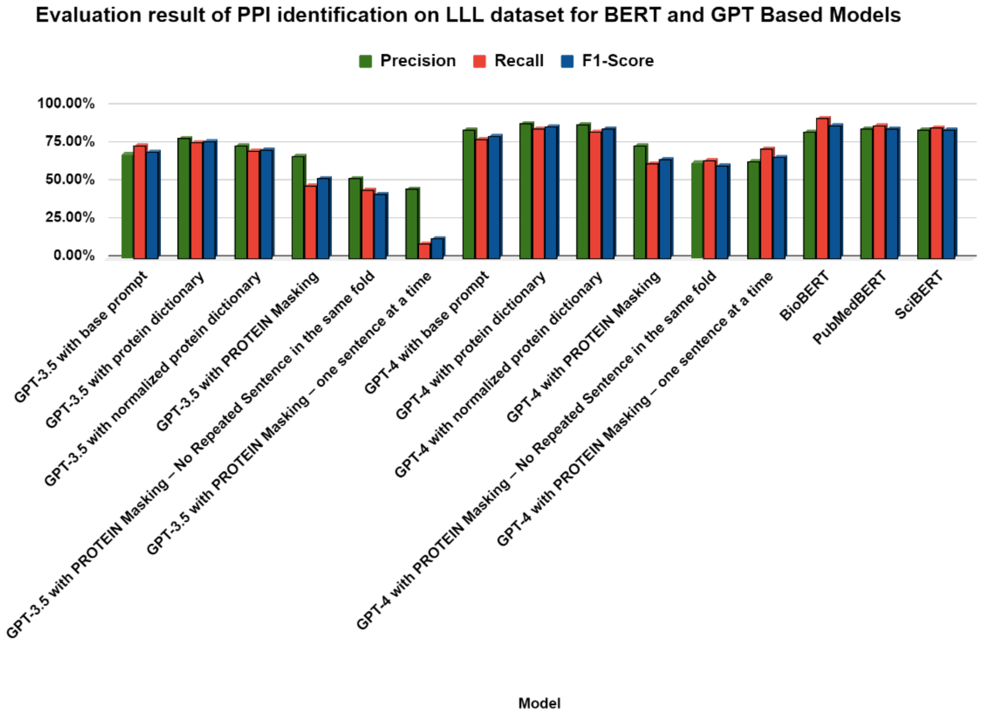

Supplementary Figure 12. Evaluation result of PPI identification on HPRD50 dataset for BERT and GPT Based Models

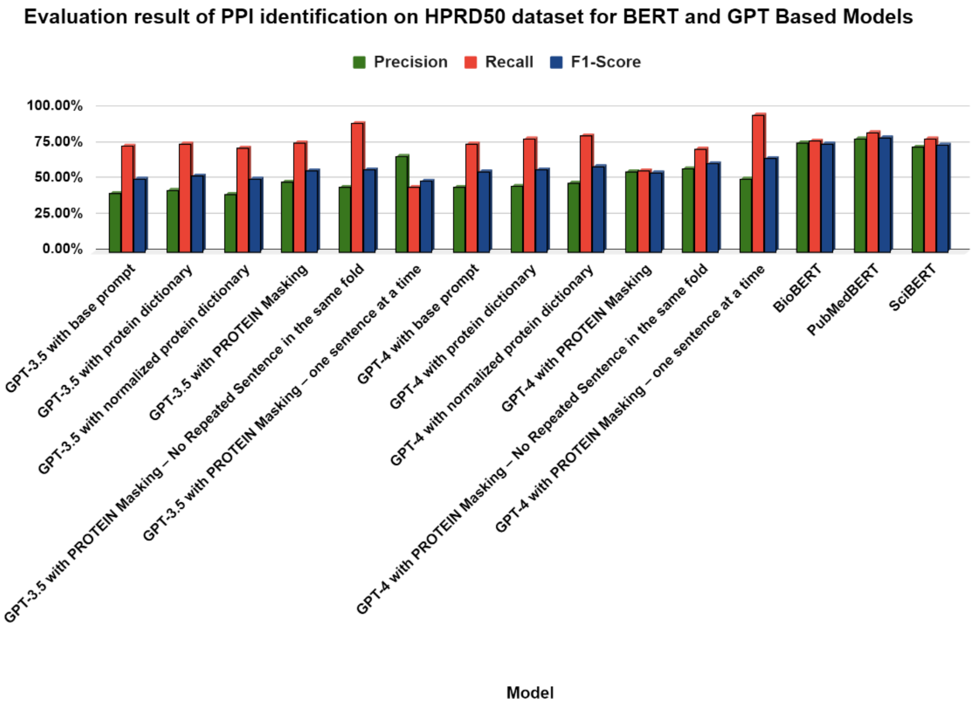

Supplementary Figure 13. Evaluation result of PPI identification on IEPA dataset for BERT and GPT Based Models

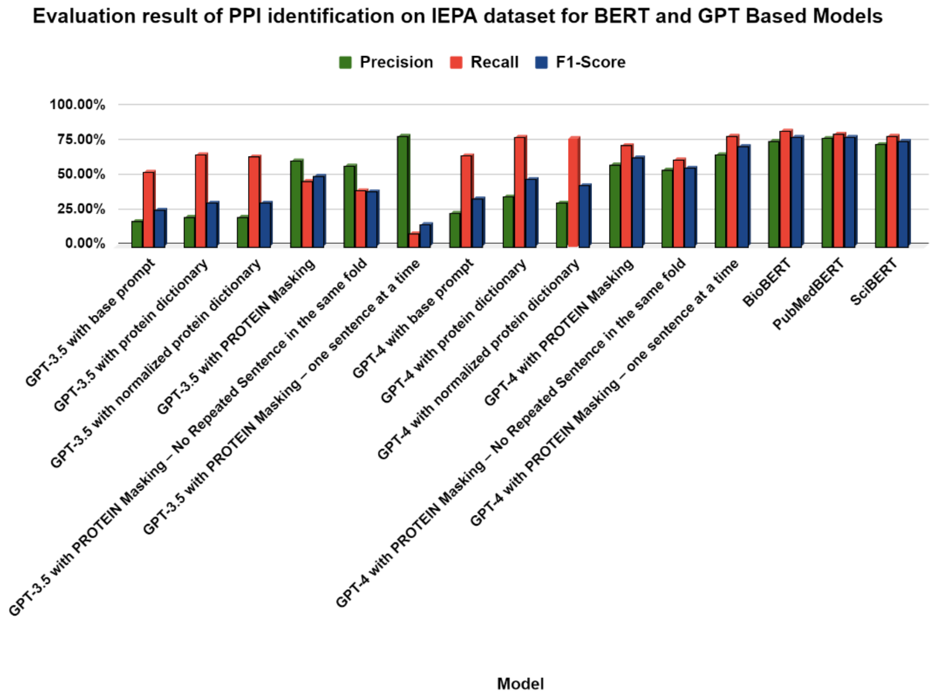

Supplementary Tables

Supplementary Table 1. Specifications of GPT models highlighting their corresponding training data, release year, structural architecture, parameter count, and context window capacity for input and output.

Model Name	Training data	Year released	Architecture	Number of Parameters	Context Window
GPT-1	Common Crawl, BookCorpus	2018	Decoder architecture of transformer with 12 layers	117 million	1024
GPT-2	- Common Crawl, BookCorpus, WebText	2019	Decoder architecture of transformer with 48 layers	1.5 billion	2048
GPT-3	Common Crawl, BookCorpus, Wikipedia, Books, Articles, and more (Up to Oct. 2019)	2020	Same model and architecture as GPT-2 with 96 layers. Variations include Davinci[#], Babbage, Curie, and Ada.	175 billion	2,049 tokens
GPT-3.5	Up to Sep. 2021	2022	A combination of three models: code-davinci-002, text-davinci-002, and text-davinci-003.	1.3 billion, 6 billion, and 175 billion	4096 tokens (Default)
GPT-4	Dataset is not not officially disclosed (Up to Sep. 2021)	2023	Fine-tuned using reinforcement learning from human feedback.	Supposedly 100 trillion	8,192 tokens (Default)

[#]Used in the current study.

Supplementary Table 2. Prompt Engineering for Base Prompt- Best Prompts from each section.

Prompt	Prompt
**P127_S1**	Consider each sentence separately and infer every pair of Protein-Protein Interactions from the provided sentences.<br>For this task, 'Proteins' and 'Genes' are synonymous.<br>If a sentence contains multiple PPI pairs, list each pair on a distinct row.<br>Please, format your results in CSV (comma-separated values) format with the following four columns: 'Sentence ID', 'Protein 1', 'Protein 2', and 'Interaction Type'. Ensure that no columns are left blank.

	Output Column Specifications:<br>'Sentence ID': The unique identifier for each sentence.<br>'Protein 1' and 'Protein 2': The entities in the sentence, representing the proteins or genes.<br>'Interaction Type': The type of interaction identified between the protein entities (e.g., 'binds to', 'inhibits').<br>If all sentences have been processed successfully, the last row should only contain the word 'Done'.<br>Each input line contains a 'Sentence ID' and corresponding 'Sentence' that is needed to be analyzed for finding PPI.<br>Here are the sentences that you need to process:
**P8_S2**	Consider each sentence separately and infer every pair of Protein-Protein Interactions from the provided sentences.<br>For this task, consider Proteins and Genes as interchangeable terms.<br>If a sentence contains multiple PPI pairs, list each pair on a distinct row.<br>Please, format your results in CSV (comma-separated values) format with the following four columns: 'Sentence ID', 'Protein 1', 'Protein 2', and 'Interaction Type'. Ensure that no columns are left blank.<br>Output Column Specifications:<br>'Sentence ID': The unique identifier for each sentence.<br>'Protein 1' and 'Protein 2': The entities in the sentence, representing the proteins or genes.<br>'Interaction Type': The type of interaction identified between the protein entities (e.g., 'binds to', 'inhibits').<br>If all sentences have been processed successfully, the last row should only contain the word 'Done'.<br>Each input line contains a 'Sentence ID' and corresponding 'Sentence' that is needed to be analyzed for finding PPI.<br>Here are the sentences that you need to process:
**P60_S3**	Consider each sentence separately and infer every pair of Protein-Protein Interactions from the provided sentences.<br>For this task, consider Proteins and Genes as interchangeable terms.<br>Provide each pair in a separate row whenever a sentence contains multiple Protein-Protein interaction pairs.<br>Please, format your results in CSV (comma-separated values) format with the following four columns: 'Sentence ID', 'Protein 1', 'Protein 2', and 'Interaction Type'. Ensure that no columns are left blank.<br>Output Column Specifications:<br>'Sentence ID': The unique identifier for each sentence.<br>'Protein 1' and 'Protein 2': The entities in the sentence, representing the proteins or genes.<br>'Interaction Type': The type of interaction identified between the protein entities (e.g., 'binds to', 'inhibits').<br>If all sentences have been processed successfully, the last row should only contain the word 'Done'.<br>Each input line contains a 'Sentence ID' and corresponding 'Sentence' that is needed to be analyzed for finding PPI.<br>Here are the sentences that you need to process:

**P10_S4**	Consider each sentence separately and infer every pair of Protein-Protein Interactions from the provided sentences.<br>For this task, consider Proteins and Genes as interchangeable terms.<br>Provide each pair in a separate row whenever a sentence contains multiple Protein-Protein interaction pairs.<br>Please format the output in CSV with the following four columns: 'Sentence ID', 'Protein 1', 'Protein 2', and 'Interaction Type'. Ensure that no columns are left blank.<br>Output Column Specifications:<br>'Sentence ID': The unique identifier for each sentence.<br>'Protein 1' and 'Protein 2': The entities in the sentence, representing the proteins or genes.<br>'Interaction Type': The type of interaction identified between the protein entities (e.g., 'binds to', 'inhibits').<br>If all sentences have been processed successfully, the last row should only contain the word 'Done'.<br>Each input line contains a 'Sentence ID' and corresponding 'Sentence' that is needed to be analyzed for finding PPI.<br>Here are the sentences that you need to process:
**P32_S5**	Consider each sentence separately and infer every pair of Protein-Protein Interactions from the provided sentences.<br>For this task, consider Proteins and Genes as interchangeable terms.<br>Provide each pair in a separate row whenever a sentence contains multiple Protein-Protein interaction pairs.<br>Please format the output in CSV with the following four columns: 'Sentence ID', 'Protein 1', 'Protein 2', and 'Interaction Type'. Ensure that no columns are left blank.<br>Output Column Specifications:<br>'Sentence ID': The unique ID for each sentence.<br>'Protein 1' and 'Protein 2': The entity pairs in the sentence representing the proteins or genes with potential PPI.<br>'Interaction Type': The type of interaction identified between the protein pairs (e.g., 'binds to', 'inhibits').<br>If all sentences have been processed successfully, the last row should only contain the word 'Done'.<br>Each input line contains a 'Sentence ID' and corresponding 'Sentence' that is needed to be analyzed for finding PPI.<br>Here are the sentences that you need to process:
**P19_S6**	Consider each sentence separately and infer every pair of Protein-Protein Interactions from the provided sentences.<br>For this task, consider Proteins and Genes as interchangeable terms.<br>Provide each pair in a separate row whenever a sentence contains multiple Protein-Protein interaction pairs.<br>Please format the output in CSV with the following four columns: 'Sentence ID', 'Protein 1', 'Protein 2', and 'Interaction Type'. Ensure that no columns are left blank.<br>Output Column Specifications:<br>The last output row should exclusively contain the word 'Done' to indicate that all the sentences have been processed successfully.<br>'Protein 1' and 'Protein 2': The entity pairs in the sentence representing the proteins or genes with potential PPI.

	'Interaction Type': The type of interaction identified between the protein pairs (e.g., 'binds to', 'inhibits').<br>If all sentences have been processed successfully, the last row should only contain the word 'Done'.<br>Each input line contains a 'Sentence ID' and corresponding 'Sentence' that is needed to be analyzed for finding PPI.<br>Here are the sentences that you need to process:
**P5_S7**	Consider each sentence separately and infer every pair of Protein-Protein Interactions from the provided sentences.<br>For this task, consider Proteins and Genes as interchangeable terms.<br>Provide each pair in a separate row whenever a sentence contains multiple Protein-Protein interaction pairs.<br>Please format the output in CSV with the following four columns: 'Sentence ID', 'Protein 1', 'Protein 2', and 'Interaction Type'. Ensure that no columns are left blank.<br>Output Column Specifications:<br>The last output row should exclusively contain the word 'Done' to indicate that all the sentences have been processed successfully.<br>'Protein 1' and 'Protein 2': The entity pairs in the sentence representing the proteins or genes with potential PPI.<br>'Interaction Type': The type of interaction identified between the protein pairs (e.g., 'binds to', 'inhibits').<br>If all sentences have been processed successfully, the last row should only contain the word 'Done'.<br>Each input line contains a 'Sentence ID' and its corresponding 'Sentence' for PPI analysis.<br>Here are the sentences that you need to process:

Prompt name format: PPromptNo_SSectionNo

Supplementary Table 3. Final Prompts for each Prompt Type.

Prompt Type	Prompt
**Base (10 fold)**	Consider each sentence separately and infer every pair of Protein-Protein Interactions from the provided sentences.<br>For this task, consider Proteins and Genes as interchangeable terms.<br>Provide each pair in a separate row whenever a sentence contains multiple Protein-Protein interaction pairs.<br>Please, format your results in CSV (comma-separated values) format with the following four columns: 'Sentence ID', 'Protein 1', 'Protein 2', and 'Interaction Type'. Ensure that no columns are left blank.<br>Output Column Specifications:<br>'Sentence ID': The unique identifier for each sentence.<br>'Protein 1' and 'Protein 2': The entities in the sentence, representing the proteins or genes.<br>'Interaction Type': The type of interaction identified between the protein entities (e.g., 'binds to', 'inhibits').<br>If all sentences have been processed successfully, the last row should only contain the word 'Done'.

	Each input line contains a 'Sentence ID' and corresponding 'Sentence' that is needed to be analyzed for finding PPI.<br>Here are the sentences that you need to process:
**With Protein Dictionary (10 fold)**	Please, format your results in CSV (comma-separated values) format with the following four columns: 'Sentence ID', 'Protein 1', 'Protein 2', and 'Interaction Type'. Ensure that no columns are left blank.<br>Output Column Specifications:<br>'Sentence ID': The unique identifier for each sentence.<br>'Protein 1' and 'Protein 2': The entities in the sentence, representing the proteins or genes.<br>'Interaction Type': The type of interaction identified between the protein entities (e.g., 'binds to', 'inhibits').<br>If all sentences have been processed successfully, the last row should only contain the word 'Done'.<br>Each input line contains a 'Sentence ID' and corresponding 'Sentence' that is needed to be analyzed for finding PPI.<br>Here are the protein names for your reference : [['KinC' 'KinD' 'sigma(A)' 'Spo0A' 'SigE' 'SigK' 'GerE' 'sigma(F)' 'sigma(G)' 'SpoIIE' 'FtsZ' 'sigma(H)' 'sigma(K)' 'gerE' 'EsigmaF' 'sigmaB' 'sigmaF' 'SpoIIAB' 'SpoIIAA' 'SigL' 'RocR' 'sigma(54)' 'E sigma E' 'YfhP' 'SpoIIAA-P' 'sigmaK' 'sigmaG' 'ComK' 'FlgM' 'sigma X' 'sigma B' 'sigma(B)' 'sigmaD' 'SpoIIID' 'sigmaW' 'PhoP~P' 'AraR' 'sigmaH' 'yvyD' 'ClpX' 'Spo0' 'RbsW' 'DnaK' 'sigmaE' 'sigma W' 'sigmaA' 'sigma(X)' 'CtsR' 'Spo0A~P' 'spoIIG' 'ydhD' 'ykuD' 'ykvP' 'ywhE' 'spo0A' 'spoVG' 'rsfA' 'cwlH' 'KatX' 'katX' 'rocG' 'yfhS' 'yfhQ' 'yfhR' 'sspE' 'yfhP' 'bmrUR' 'ydaP' 'ydaE' 'ydaG' 'yfkM' 'sigma F' 'cot' 'sigK' 'cotD' 'sspG' 'sspJ' 'hag' 'comF' 'flgM' 'ykzA' 'CsbB' 'nadE' 'YtxH' 'YvyD' 'bkd' 'degR' 'cotC' 'cotX' 'cotB' 'sigW' 'tagA' 'tagD' 'tuaA' 'araE' 'sigmaL' 'spo0H' 'sigma G' 'sigma 28' 'sigma 32' 'spoIVA' 'PBP4*' 'RacX' 'YteI' 'YuaG' 'YknXYZ' 'YdjP' 'YfhM' 'phrC' 'sigE' 'ald' 'kdgR' 'sigX' 'ypuN' 'clpC' 'ftsY' 'gsiB' 'sigB' 'sspH' 'sspL' 'sspN' 'tlp']]<br>Here are the sentences that you need to process:
**With Normalized Protein Dictionary (10 fold)**	Please, format your results in CSV (comma-separated values) format with the following four columns: 'Sentence ID', 'Protein 1', 'Protein 2', and 'Interaction Type'. Ensure that no columns are left blank.<br>Output Column Specifications:<br>'Sentence ID': The unique identifier for each sentence.<br>'Protein 1' and 'Protein 2': The entities in the sentence, representing the proteins or genes.<br>'Interaction Type': The type of interaction identified between the protein entities (e.g., 'binds to', 'inhibits').<br>If all sentences have been processed successfully, the last row should only contain the word 'Done'.<br>Each input line contains a 'Sentence ID' and corresponding 'Sentence' that is needed to be analyzed for finding PPI.<br>Here are the normalized protein names for your reference : [['kinc' 'kind' 'sigmaa' 'spo0a' 'sige' 'sigk' 'gere' 'sigmaf' 'sigmag' 'spoiie' 'ftsz' 'sigmah' 'sigmak' 'esigmaf' 'sigmab' 'spoiiab' 'spoiiaa' 'sigl' 'rocr' 'sigma54' 'esigmae' 'yfhp' 'spoiiaa-p' 'comk' 'flgm' 'sigmax' 'sigmad' 'spoiiid' 'sigmaw' 'phop~p' 'arar' 'yvyd' 'clpx' 'spo0' 'rbsw' 'dnak' 'sigmae' 'ctsr' 'spo0a~p' 'spoiig' 'ydhd' 'ykud' 'ykvp' 'ywhe' 'spovg' 'rsfa' 'cwlh' 'katx' 'rocg' 'yfhs' 'yfhq' 'yfhr' 'sspe' 'bmrur' 'ydap' 'ydae' 'ydag' 'yfkm' 'cot' 'cotd' 'sspg' 'sspj'

	'hag' 'comf' 'ykza' 'csbb' 'nade' 'ytxh' 'bkd' 'degr' 'cotc' 'cotx' 'cotb' 'sigw' 'taga' 'tagd' 'tuaa' 'arae' 'sigmal' 'spo0h' 'sigma28' 'sigma32' 'spoiva' 'pbp4*' 'racx' 'ytei' 'yuag' 'yknxyz' 'ydjp' 'yfhm' 'phrc' 'ald' 'kdgr' 'sigx' 'ypun' 'clpc' 'ftsy' 'gsib' 'sigb' 'ssph' 'sspl' 'sspn' 'tlp']]<br>Here are the sentences that you need to process:
**With PROTEIN masking (10-fold)**	Consider each sentence separately and infer Protein-Protein Interaction for the protein entity pairs PROTEIN1-PROTEIN2 from the provided sentences. Do not consider any other PROTEIN pairs in the sentence. In each sentence, original protein or gene names have been substituted with 'PROTEIN1', 'PROTEIN', and 'PROTEIN' placeholders. The placeholders used may represent a variety of proteins or genes, differing with each sentence.<br>Please, format your results in CSV (comma-separated values) with only two columns: 'Sentence ID' and 'PPI'. Do not include the original sentences or any explanation in the output.<br>Output Column Specifications:<br>'Sentence ID': The unique identifier for each sentence.<br>'PPI': Record your findings as 'TRUE' if there is a demonstrable interaction between PROTEIN1 and PROTEIN2, and 'FALSE' if there is none.<br>If all sentences have been processed successfully, the last row should only contain the word 'Done'.<br>Each input line contains a 'Sentence ID' and corresponding 'Sentence' that is needed to be analyzed for finding PPI.<br>Here are the sentences that you need to process:
**With PROTEIN masking (Nfold)**	Same as With PROTEIN masking (10-fold)
**With PROTEIN masking (One sentence at a time)**	Infer Protein-Protein Interaction for the protein entity pairs PROTEIN1-PROTEIN2 from the provided sentence. Do not consider any other PROTEIN pairs in the sentence. Original protein or gene names have been substituted with 'PROTEIN1', 'PROTEIN', and 'PROTEIN' placeholders.<br>Output Specification: Record your findings as 'TRUE' if there is a demonstrable interaction between PROTEIN1 and PROTEIN2, and 'FALSE' if there is none.<br>Here is the sentence that you need to process:

# 10 fold - Sentences in each fold here are the same as BERT, Nfold – The same sentence with different positional PROTEIN masking is not present in the same fold.

Supplementary Table 4. PPI identification macro scores of the BERT-based models on LLL

Model Name	Macro Precision	Macro Recall	Macro F1 Score
BioBERT	86.80	85.92	85.66
PubMedBERT	86.44	85.08	84.79
SciBERT	86.16	82.95	83.52

Supplementary Table 5. PPI identification macro scores of the BERT-based models on IEPA

Model Name	Macro Precision	Macro Recall	Macro F1 Score
BioBERT	81.55	83.21	78.81
PubMedBERT	82.20	81.56	81.15
SciBERT	79.08	79.55	78.72

Supplementary Table 6. PPI identification macro scores of the BERT-based models on HPRD50

Model Name	Macro Precision	Macro Recall	Macro F1 Score
BioBERT	75.85	72.39	71.72
PubMedBERT	82.60	80.82	80.78
SciBERT_scivocab_cased	71.48	69.88	68.74
